# lncRNA PRADX is a Mesenchymal Glioblastoma Biomarker for Cellular Metabolism Targeted Therapy

**DOI:** 10.3389/fonc.2022.888922

**Published:** 2022-04-29

**Authors:** Can Xu, Jixing Zhao, Jia Song, Menglin Xiao, Xiaoteng Cui, Lei Xin, Jianglong Xu, Yuhao Zhang, Kaikai Yi, Biao Hong, Fei Tong, Shaohui Tian, Yanli Tan, Chunsheng Kang, Chuan Fang

**Affiliations:** ^1^ School of Clinical Medicine, Hebei University, Department of Neurosurgery, Affiliated Hospital of Hebei University, Baoding, China; ^2^ Hebei Key Laboratory of Precise Diagnosis and Treatment of Glioma, Baoding, China; ^3^ Department of Neurosurgery, Tianjin Medical University General Hospital, Laboratory of Neuro-oncology, Tianjin Neurological Institute, Tianjin, China; ^4^ Key Laboratory of Post-Neurotrauma Neuro-Repair and Regeneration in Central Nervous System, Ministry of Education and Tianjin City, Tianjin, China; ^5^ School of Basic Medical Sciences, Hebei University, Baoding, China

**Keywords:** mesenchymal GBM, lncRNA, RUNX1-CBFβ complex, STAT3 pathway, energy metabolism

## Abstract

Glioblastoma (GBM) is the most common and lethal type of primary malignant central nervous system (CNS) tumor with an extremely poor prognosis, and the mesenchymal subtype of GBM has the worst prognosis. Here, we found that lncRNA PRADX was overexpressed in the mesenchymal GBM and was transcriptionally regulated by RUNX1-CBFβ complex, overexpressed PRADX suppressed BLCAP expression *via* interacting with EZH2 and catalyzing trimethylation of lysine 27 on histone H3 (H3K27me3). Moreover, we showed that BLCAP interacted with STAT3 and reduced STAT3 phosphorylation, overexpressed PRADX activated STAT3 phosphorylation, and promoted ACSL1 expression *via* suppressing BLCAP expression, accelerating tumor metabolism. Finally, we determined that combined of ACSL1 and CPT1 inhibitors could reverse the accelerated cellular metabolism and tumor growth induced by PRADX overexpression *in vivo* and *in vitro*. Collectively, PRADX/PRC2 complex activated the STAT3 pathway and energy metabolism in relation to mesenchymal GBM progression. Furthermore, our findings provided a novel therapeutic strategy targeting the energy metabolism activity of GBM.

## Introduction

Long non-coding RNAs (lncRNAs) are defined as a versatile class of RNA transcripts, longer than 200 nucleotides, lacking protein-coding capacity (1). Accumulating evidence suggests that lncRNA dysregulation plays a pivotal role in the onset and progression of a broad spectrum of cancers, including glioblastoma (GBM) (2, 3). With the increasing appreciation of lncRNAs’ indispensable mechanistic involvement and the subsequent technological advancements in biomedical research, novel lncRNAs are constantly being discovered in relation to GBM pathology. Emerging studies have shown that lncRNAs act as the key regulators of the gene expression pattern not only under the normal physiological condition but also in the pathogenesis and progression of GBM (4–6). Furthermore, aberrant lncRNA expression profiles in GBM patients correlate with their respective cancer malignancies and molecular subtypes, which have important clinical implications in the diagnosis and progression of GBM (7–9). lncRNAs have also been reported to participate in chromatin dynamics and gene expression regulation by interacting with key epigenetic regulatory factors (10, 11). In the case of GBM, studies have shown that lncRNAs modulate chromatin remodeling in association with polycomb repressive complex 2(PRC2) (12–14). PRC2 regulates the transcriptional repression by hyper-methylating histone H3K27 through its methyltransferase activity (15, 16). Notably, PRC2 subunits encoding genes have been found dysregulated in a variety of cancers, including GBM. Hence, small-molecule inhibitors restoring PRC2 function have entered clinical trials for cancers (17, 18).

GBM is the most aggressive and frequent adult brain tumor with only a 5.1% five-years survival rate, and the mean survival time of GBM patients is about 15 months, even with multimodal therapy (19). Due to the extremely unfavorable prognosis and unknown underlying mechanism, the Cancer Genome Atlas (TCGA) has classified GBM into four major molecular subtypes- proneural, neural, mesenchymal, and classical, according to the gene expression data from three different platforms (20). Later in 2017, TCGA published the modified GBM classification based on the immune microenvironment of the tumor. After excluding the potential environmental risk factors, GBM has been reclassified into three molecular subtypes, namely proneural, mesenchymal, and classical. Interestingly, patients with mesenchymal subtypes show a tendency toward higher transcriptional heterogeneity and worse survival outcomes compared to proneural and classical subtypes (21). Sumazin et al. have exploited the TCGA GBM database to establish a post-transcriptional regulatory network involving microRNAs(miRNA) through the HERMES multivariate analysis method. The study has found that 6 genes, including runt-related transcription factor 1 (RUNX1) play a key role in the occurrence and development of GBM pathology (22). In 2018, we reported the construction of a competitive endogenous RNA (ceRNA) regulation network linking the protein-coding functional mRNA to the mechanistically related lncRNAs based on the integrated omics analysis, which has further confirmed that dysregulation of RUNX1 is a key pathological inducer of the mesenchymal GBM subtype (23). These findings have also been recapitulated in both *in vivo* and *in vitro* GBM models, including patients’ samples (24).

Our previous study identified a novel lncRNA ENST00000449248.1, which we named PRADX, due to its association with PRC2 and DEAD-box helicase 5 (DDX5). We confirmed that PRADX could serve as a potential prognostic indicator of GBM and colon adenocarcinoma (COAD). Previously we have shown that the recruitment of the PRC2/DDX5 complex increases the occupancy of H3K27me3 at the UBX domain protein 1 (UBXN1) gene promoter region, thereby suppressing UBXN1 expression and promoting NF-κB activity (25). In the present study, we furthered the investigation of the transcriptional regulatory mechanism of PRADX to reveal its oncogenic role in the mesenchymal GBM *via* promoting energy metabolism.

## Material and Methods

### Cell Lines and Cell Transfection

Culture methods of the primary cell lines (N33, N9, and TBD0220) used in this study have been previously reported (26). All the cells were cultured in Dulbecco’s Modified Eagle’s Medium (DMEM/F12, 1:1; Gibco) containing 10% fetal bovine serum (FBS). PRADX lentiviral vector was transduced according to the manufacturer’s instructions (Genechem, Shanghai, China), Small interfering RNAs (siRNAs) targeting PRADX, EZH2, RUNX1, CBFβ, and BLCAP were purchased from GenePharma (Suzhou, China), RUNX1 plasmid was purchased from Ibsbio (Shanghai, China). See **Supplementary Table S4** for details.

### Cell Counting Kit 8 and Colony Formation Assays

CCK-8 (Dojindo, Japan) assay was performed to evaluate the cell viability by measuring the absorbance at 450 nm (OD450) using BioTek Gen5 Microplate Reader (BioTek Instruments, USA). A colony formation assay was performed to evaluate the colony formation ability of the specific cancer types, following the method described in our previous study (25).

### Seahorse XFe Extracellular Flux Analysis (Mitochondrial Stress Test)

Extracellular flux analysis was performed to evaluate cellular metabolism indexes, including oxygen consumption rate (OCR), basal respiration, proton leak, and ATP production. The experiments were performed on a Seahorse XF24 Analyzer (Seahorse Bioscience) as described elsewhere (26).

### Western Blotting and Co-Immunoprecipitation

Total cells were lysed using RIPA buffer with proteinase and phosphatase inhibitor cocktails (Selleck Chemicals, Shanghai, China). After transferring the protein bands onto the polyvinylidene fluoride (PVDF) membrane, the membrane was incubated with the respective primary antibodies at 4°C overnight, followed by horseradish peroxidase (HRP)-conjugated secondary antibody incubation for 1 h at room temperature. The protein bands were observed using a chemiluminescence reagent (ECL) (Boster, Wuhan, China). For co-IP, IP lysis buffer (Beyotime Biotechnology) was used to lyse the cells. For immunoprecipitation, 40μl of protein-A/G agarose beads (Millipore) and 5μg anti-STAT3 or anti-BLCAP antibody were incubated with the total cell lysates at 4°C overnight. The immunoblots were then washed five times with 1X phosphate-buffered saline (PBS) buffer for the downstream signal intensity detection processes.

### Chromatin Immunoprecipitation

The Millipore Magna ChIP TM A/G kit (catalog # 17-10085) was used to perform the ChIP experiment. A total of 6×106 cells was cross-linked using 1% formaldehyde solution for 10 min followed by 10X glycine neutralization for 5 min at room temperature. The cell lysate was then sonicated to generate the sheared DNA fragments of 200-1000 bp in size. After that, 5μg of each of the target antibodies were immunoprecipitated with an equal amount of chromatin fraction at 4°C overnight. The products were incubated with magnetic Protein A/G beads and quantitated by qPCR assay. The antibodies and primers are listed in **Supplementary Table S3**.

### RNA Isolation and Real-Time Quantitative Reverse-Transcription PCR

TRIzol reagent (Invitrogen, USA) was used to extract the total RNA from cultured cells. 1-5ug of the total RNA was used for cDNA synthesis using the PrimeScript RT Reagent Kit (Takara, Japan). SYBR Green reaction mix (Takara, Japan) was used to perform the RT-qPCR assay with CFX96 Touch Real-Time PCR Detection System (Bio-Rad, USA). The primers are listed in **Supplementary Table S2**.

### Confocal Immunofluorescence Microscopy

The cells were cultured on coverslips using 24-well plates overnight and then fixed with 4% formaldehyde. Cells were permeabilized with 0.5% Triton-X100 (ThermoFisher, USA) for 20min at room temperature followed by blocking with 5% bovine serum albumin (BSA). The primary antibodies were incubated overnight at 4°C followed by corresponding secondary antibody incubation for 1 h at room temperature. The 4’-6-diamidino-2-phenylindole (DAPI, Molecular Probes, D1306) was used to counterstain the nucleus and phalloidin for the cytoskeleton. The images were captured by Zeiss 510 META laser scanning confocal microscope.

### Dual-Luciferase Report Assay

The cells were cultured in a 24-well plate for 24h followed by co-transfection of 0.5-2.0μg of PRADX plasmid and 25ng of pRL-TK (renilla luciferase reporter vector) reporter plasmids using jetPRIME kit. After 24–36 h, cells were harvested to observe the luciferase activity by the Dual-Glo Luciferase system (Promega) with the normalization to pRL-TK.

### *In Vivo* Xenograft Mouse Models

BALB/c nude mice were purchased from Beijing Vital River Laboratory Animal Technology. PRADX overexpressed or scrambled TBD0220 cells were pre-transfused with luciferase lentiviral particles. The stereotactic injection was applied to inject the cancer cells (3 × 10^5^ cells per mouse in 3μL PBS) into the intracranial space. The bioluminescence imaging was captured by the IVIS Lumina Imaging System (Xenogen) after 7,14,21, and 28d of injection. Intact brains were carefully extracted after euthanasia and then embedded into the paraffin section for IHC analysis.

### Immunohistochemistry Staining

Tumor sections were deparaffinized in xylene followed by gradient ethanol hydration, and antigen recovery was carried out in ethylene diamine tetraacetic acid (EDTA) solution. Tumor sections were incubated with primary antibodies at 4°C overnight, followed by HRP-conjugated secondary antibody probing for 1h at room temperature. After the diaminobenzidine (DAB) staining, the sections were observed under a light microscope. The antibodies are listed in **Supplementary Table S1**.

### Statistical analysis

All the statistical analyses were performed using GraphPad Prism 8. The transcriptional and clinical data were obtained from CGGA (http://www.cgga.org.cn/) and TCGA (https://tcga-data.nci.nih.gov/tcga/tcgaDownload.jsp) databases. Gene set enrichment (GSEA) analysis was performed with GSEA_4.1.0 software. Clustered heatmap analysis was performed using the online bioinformatics tools (http://bioinformatics.com.cn/). The error bars in the figures represent the mean ± standard deviation (SD) from at least three independent experiments. A P value of <0.05 was considered statistically significant.

## Results

### PRADX is a Biomarker of Mesenchymal GBM

**Figure 1:**
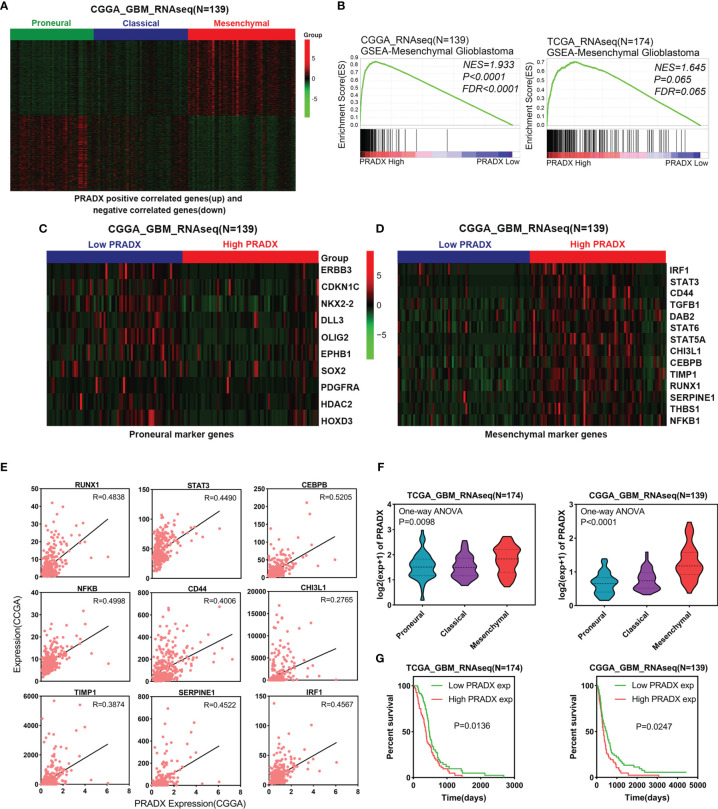
PRADX expression correlates with mesenchymal GBM. **(A)** Clustered heat map showing that PRADX positively correlated genes (top 500) and negatively correlated genes (top 500) expression profiles in the CGGA database. **(B)** GSEA analysis of mesenchymal GBM gene expression between PRADX high and low expression groups in TCGA and CGGA databases. **(C, D)** Clustered heat map showing that proneural and mesenchymal marker genes expression profiles in PRADX high expression and low expression patients. **(E)** The correlation between PRADX and mesenchymal marker genes in the CGGA database. **(F)** Violin plots exhibiting PRADX expression in different molecular subtypes of GBM in TCGA and CGGA databases. **(G)** Kaplan-Meier curve showing patients’ survival times between PRADX high expression and low expression groups.

To investigate whether PRADX was related to the mesenchymal subtype of GBM, we searched the top 500 positively and top 500 negatively correlated genes of PRADX in the CGGA database and performed clustered heat map analysis. The results showed that the positively correlated genes were enriched in patients with the mesenchymal subtype, while the negatively correlated genes were clustered in the proneural subtype (**Figure 1A**). In addition, PRADX highly expressed patients with a high expression of mesenchymal marker genes and low expression of proneural marker genes (**Figures 1C, D**). GSEA analysis was performed using TCGA and CGGA databases by dividing the patients into high and low expression groups based on their respective PRADX expression profiles. Moreover, patients with high PRADX expression were enriched in the mesenchymal subtype (**Figure 1B**). The Pearson correlated coefficient also indicated that PRADX is positively correlated with mesenchymal GBM marker genes (**Figure 1E**). One-way ANOVA analysis revealed that PRADX expression was significantly higher in patients with mesenchymal subtype from both TCGA and CGGA databases (**Figure 1F**). Furthermore, we investigated the survival influence of PRADX in GBM according to TCGA and CGGA databases, Kaplan-Meier survival curve indicated that PRADX upregulation was associated with a shorter survival time in GBM patients (**Figure 1G**). These findings cumulatively suggest that PRADX is a potential biomarker of mesenchymal subtype, and its increased expression is correlated with the poor prognosis of GBM patients.

### PRADX is Transcriptionally Upregulated by RUNX1-CBFβ Complex

**Figure 2:**
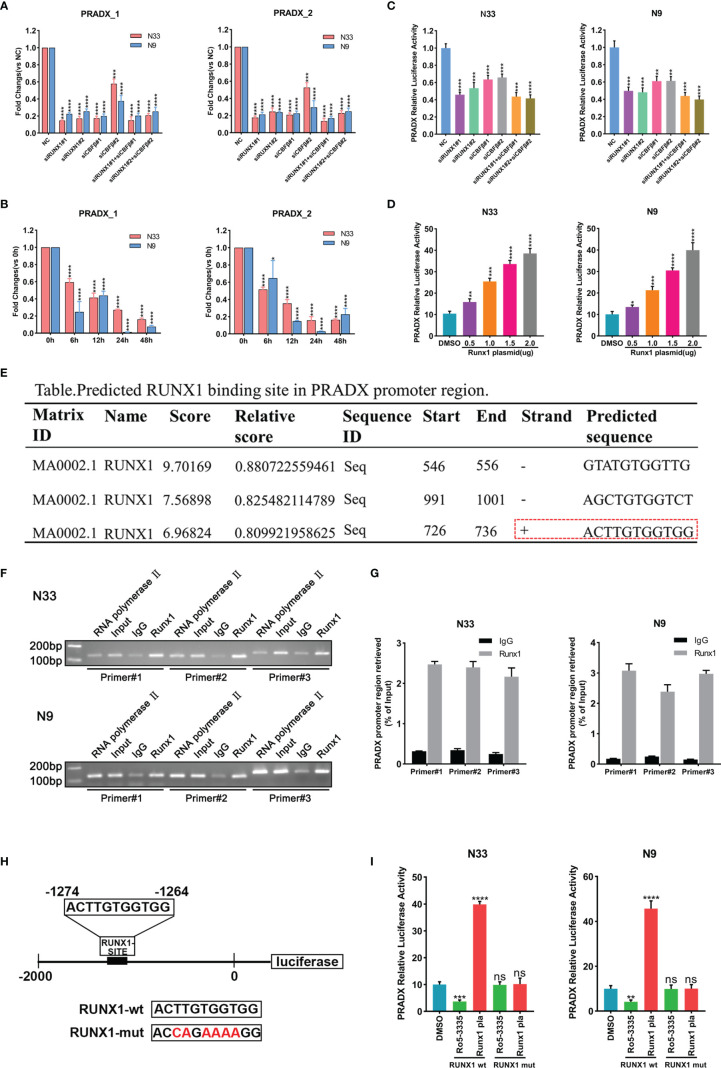
PRADX was transcriptionally regulated by the RUNX1-CBFβ complex. **(A)** qRT-PCR assay exhibiting PRADX expressions after treating with RUNX1 and/or CBFβ siRNA. **(B)** qRT-PCR showing PRADX expressions after treating with RO5-3335 for different times. **(C, D)** Dual-luciferase report assay showing the transcriptional activity of PRADX after treating with RUNX1, CBFβ siRNA, or RUNX1 expression plasmid. **(E)** The binding site of RUNX1 in the PRADX promoter region as predicted by the JASPAR tool. **(F, G)** ChIP-PCR and ChIP-qPCR analyses show RUNX1 binds to the predicted binding site on the PRADX promoter. **(H, I)** RUNX1 binding site mutated plasmid construction for dual-luciferase reporter assay to compare PRADX transcription activities following RO5-3335 treatment or RUNX1 expression plasmid transfection in the indicated groups. Data are represented as mean ± standard deviation (SD); n = 3 independent experiments. ****P <0.00001, ***P <0.0001, **P <0.001, *P <0.05. ns: no significance.

RUNX1 has already been reported as a mesenchymal biomarker in the previous study and is associated with a shorter survival time in GBM patients (23, 24). Although RUNX1 is a nuclear transcription factor, the CBFβ chaperone is essential for RUNX1’s transcriptional function (27, 28). To investigate if PRADX was regulated by RUNX1 and CBFβ expression, We transfected N33 and N9 cells with RUNX1 and CBFβ siRNA alone or in combination, and designed two pairs of PCR primers of PRADX, the subsequent qRT-PCR assay revealed PRADX level was significantly reduced (**Figure 2A**), Consistently, N33 and N9 cells were treated with RUNX1-CBFβ binding inhibitor RO5-3335(25µM) for 0, 6, 12, 24, and 48h, PRADX expression was reduced after RO5-3335 treatment in a time-dependent manner (**Figure 2B**). Furthermore, we constructed a luciferase reporter plasmid with a PRADX promoter region in the PGL4.10 vector. Dual-luciferase report assay consistently showed that PRADX transcription activity was significantly reduced after RUNX1 and CBFβ siRNA transfection (**Figure 2C**). In contrast, RUNX1 overexpression increased PRADX transcription activity in a dose-dependent manner (**Figure 2D**). These findings demonstrated that PRADX was transcription regulated by the RUNX1-CBFβ complex. All the siRNAs, lentivirus, and plasmid transfection efficiency were confirmed in Supplementary Figure S1 (**Figure S1**).

Next, we sought to understand the specific mechanism of RUNX1-CBFβ-mediated transcriptional regulation of PRADX. Firstly, we predicted the RUNX1 binding site in the PRADX promoter region based on Jaspar (http://jaspar.genereg.net/) and found only one predicted forward strand sequence (**Figure 2E**). Then, we designed 3 pairs of ChIP primers based on the predicted binding sequence. The ChIP-qPCR analysis confirmed RUNX1 could bind to the predicted binding site on the PRADX promoter region (**Figures 2F, G**). To reconfirm this finding, we constructed another dual-luciferase reporter plasmid for the PRADX promoter region but with site-directed mutation of the RUNX1 binding site (**Figure 2H**). Dual-luciferase reporter assays revealed that PRADX transcriptional activity was independent of either RUNX1 overexpression or its binding inhibition to CBFβ (RO5-3335 treatment) after RUNX1 binding site mutation (**Figure 2I**). Taken together, these findings demonstrated that RUNX1-CBFβ complex-mediated transcriptional regulation of PRADX expression is modulated by the RUNX1 binding at the specific promoter sequence of PRADX.

### PRADX Overexpression Suppresses BLCAP Expression *via* Interacting With EZH2 and Recruiting H3K27me3

**Figure 3:**
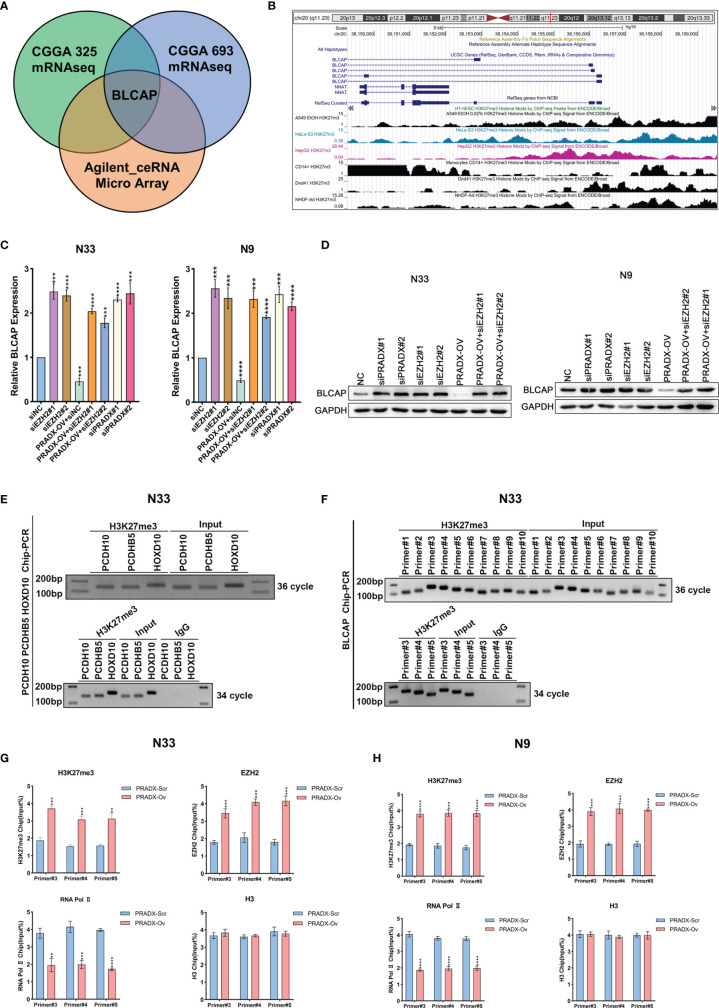
The interaction of PRADX and EZH2 silences BLCAP expression *via* recruiting H3K27me3 at the BLCAP promoter. **(A)** BLCAP was the most negatively correlated TSG with PRADX in the CGGA database and Agilent ceRNA microarray analysis. **(B)** H3K27me3 binding peaks on the BLCAP promoter region in different cell lines as per the UCSC webtool. **(C, D)** qRT-PCR and western blotting analyses revealing BLCAP expressions after PRADX overexpression or EZH2 siRNA treatment. **(E)** Application of the ChIP primers of the three TSGs reported in the literature to verify the target protein binding at the chromatin level. **(F)** ChIP-PCR showed H3K27me3 enrichment at the BLCAP promoter region. **(G, H)** ChIP-qPCR exhibits the occupancy levels of H3K27me3, EZH2, RNA polymerase II, and H3 at the BLCAP promoter region in scrambled and PRADX overexpressed cells. The values in **(C, G, H)** are represented as mean ± SD (n = 3). **P < 0.01, ***P < 0.001, ****P <0.0001.

Our previous studies have proved that PRADX mediates UBXN1 gene silencing by recruiting H3K27me3 *via* interacting with EZH2 (25). To further explore the carcinogenic mechanisms of PRADX in mesenchymal GBM, we aimed to explore new target genes and their carcinogenic pathways. We successfully overexpressed PRADX in N33 cells and performed RNA microarray analysis. Together with TCGA and CGGA cohort analysis, we found out that PRADX negatively regulated the tumor suppressor gene (TSG) BLCAP in GBM (**Figure 3A**). TSG database was downloaded from TSG (https://bioinfo.uth.edu/TSGene/) website. We then searched for an H3K27me3 binding peak in the BLCAP promoter regions of different cell lines at the UCSC (http://genome.ucsc.edu/) website (**Figure 3B**). Next, we explored if PRADX could negatively regulate BLCAP expression *via* its interaction with EZH2. To do so, we performed western blotting and qRT-PCR analyses in EZH2 siRNA transfected, PRADX siRNA transfected, and PRADX overexpressed plus EZH2 siRNA transfected N33 and N9 cells. The results demonstrated that PRADX overexpression suppressed BLCAP expression, PRADX overexpressed plus EZH2 knockdown also increased BLCAP expression (**Figures 3C, D**). Then, we conducted a ChIP experiment using primers against 3 TSG genes (PCDH10, PCDHB5, and HOXD10) (29) to verify the chromatin binding of the target proteins (**Figure 3E**). Furthermore, we designed 10 pairs of ChIP primers for BLCAP and selected 3 pairs of the most efficient primers by ChIP-PCR in N33 cells (**Figure 3F**). ChIP-qPCR analysis showed PRADX overexpression could increase the recruitment of both H3K27me3 and EZH2 at the BLCAP promoter region, whereas reduced the occupancy of RNA Polymerase II pSer2. However, there were no significant changes in total H3 levels in both N33 and N9 cells, indicating transcriptional repression (**Figures 3G, H**). In summary, these findings demonstrate that PRADX overexpression suppresses BLCAP expression *via* interacting with EZH2 and recruiting H3K27me3 at the BLCAP promoter.

### PRADX overexpression Activates the Phosphorylation of STAT3 *via* Suppressing BLCAP Expression

**Figure 4:**
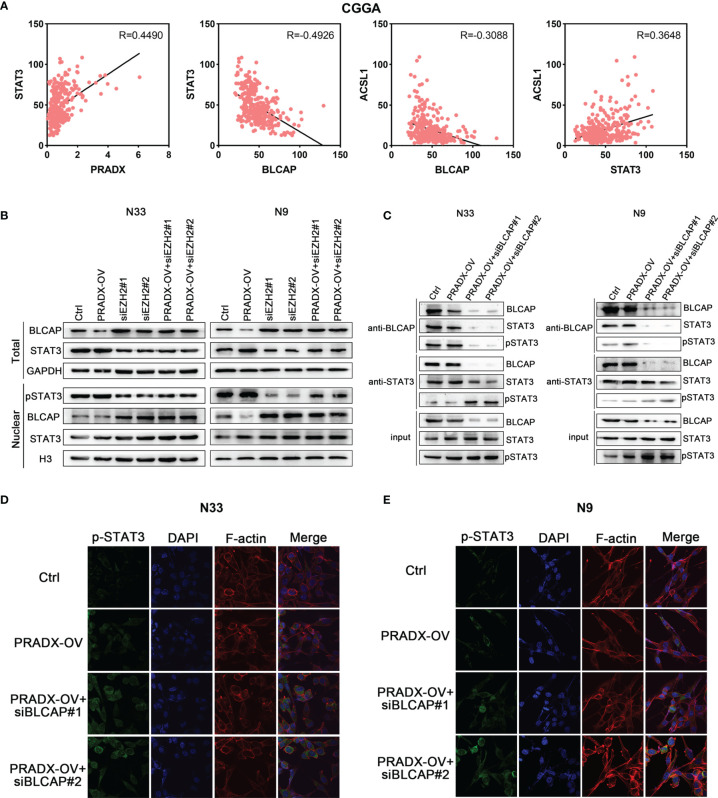
BLCAP interacts with STAT3, and overexpression of PRADX activates the phosphorylation of STAT3 *via* suppressing BLCAP expression. **(A)** The correlation between STAT3, PRADX, ACSL1, and BLCAP mRNA expression levels in the CGGA dataset. R, Pearson r value. **(B)** Western blotting results indicate the total protein levels of BLCAP, STAT3, and GAPDH and nuclear protein levels of p-STAT3, BLCAP, STAT3, and H3 in indicated groups. **(C)** Co-IP assays were performed with anti-BLCAP or anti-STAT3 antibodies followed by immunoblotting with BLCAP, STAT3, and p-STAT3 antibodies. **(D, E)** IF staining shows p-STAT3 protein expression and localization in the indicated groups. In **(B, C)**, GAPDH and H3 served as a positive control.

STAT3 is an important transcription factor and is well known for its roles in cancer proliferation, survival, invasion, and immunosuppression (30, 31) is also implicated in mesenchymal GBM (27). While BLCAP is reported as a novel STAT3 interaction partner in bladder cancer (32), and A-to-I editing of BLCAP mRNA loses the inhibitory regulation of STAT3 activation in cervical cancer (33). But the mechanistic cross-talks between BLCAP and STAT3 expressions in GBM remain unclear. Pearson’s correlation coefficient analysis showed PRADX expression was positively correlated with STAT3 level (R=0.4490), while BLCAP expression was negatively correlated with STAT3 (R=-0.4926) in the CGGA database (**Figure 4A**). Western blot analysis showed that tumor cells overexpressing PRADX had reduced levels of BLCAP and increased nuclear levels of phosphor(p)-STAT3. However, EZH2 knockdown with or without PRADX overexpression in tumor cells revealed increased levels of BLCAP and reduced nuclear levels of p-STAT3 (**Figure 4B**). These findings thus suggest that PRADX overexpression activates STAT3 phosphorylation *via* suppressing BLCAP expression.

To further explore whether BLCAP interacted with STAT3 and inhibited STAT3 phosphorylation in GBM cells, we performed a co-IP assay using an anti-BLCAP antibody in N33 and N9 total cell lysates. The results confirmed that BLCAP interacted with STAT3, and there was no change in total Stat3 levels in the nucleus and only the p-Stat3 levels change in response to PRADX overexpression and EZH2 knockdown overexpression of PRADX along with BLCAP knockdown increased STAT3 phosphorylation levels in the anti-STAT3 IP product (**Figure 4C**). Moreover, IF analysis revealed that BLCAP knockdown with or without PRADX overexpression increased nuclear p-STAT3 levels in N33 and N9 cells (**Figures 4D, E**), these findings indicate that BLCAP non-functionally interacts with STAT3, and PRADX overexpression induces STAT3 phosphorylation *via* suppressing BLCAP expression.

### PRADX Overexpression Promotes STAT3 Downstream Genes’ Expression, Including ACSL1

Recent studies have shown that the STAT3 signaling pathway promotes tumor fatty acid metabolism (34). Acyl-CoA synthetase long-chain family member 1 (ASCL1) promotes the biosynthesis of Acyl-CoA, regulates mitochondrial respiration, β-oxidation, and ATP production by regulating the activity of carnitine palmitoyltransferase 1 (CPT1), and affects fatty acid metabolism (35). Our previous study has demonstrated that ACSL1 is an important ceRNA in mesenchymal GBM (23). Pearson’s correlation coefficient analysis showed ACSL1 was positively correlated with STAT3 level (R=0.3648), negatively correlated with BLCAP level (R=-0.3088) (**Figure 4A**).

**Figure 5:**
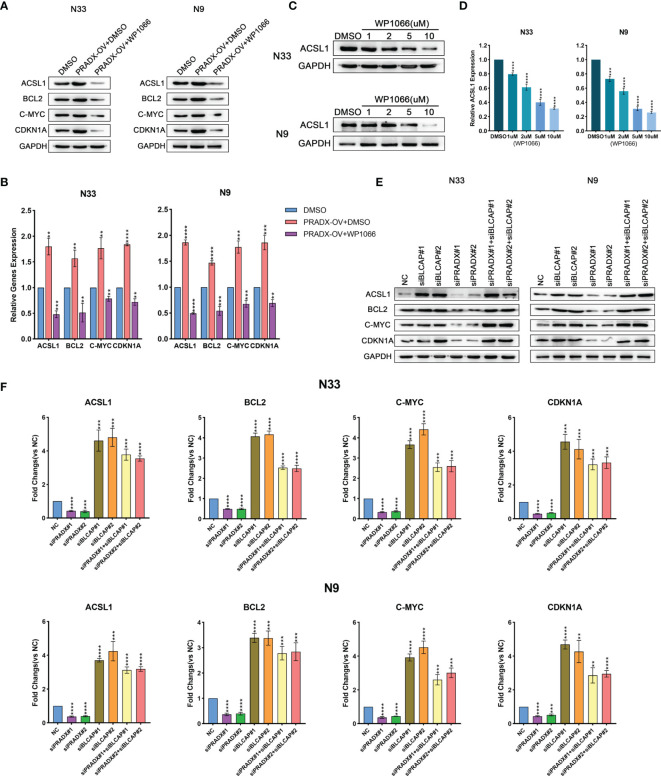
PRADX activates the STAT3 pathway *via* suppressing BLCAP. **(A, B)** qRT-PCR and western blotting analyses showing ACSL1, BCL2, c-MYC, and CDKN1A mRNA and protein expression levels in PRADX overexpressed, PRADX overexpressed plus WP1066 and DMSO groups. **(C, D)** qRT-PCR and western blot analysis showed WP1066 treatment decreased ACSL1 mRNA and protein levels in a dose-dependent manner. GAPDH served as a positive control. **(E, F)** qRT-PCR and western blot analyses demonstrating ACSL1, BCL2, c-MYC, and CDKN1A mRNA and protein expression levels in BLCAP and/or PRADX knockdown and NC groups. The values in **(B, D, F)** are represented as mean ± SD (n = 3). **P < 0.01, ***P < 0.001, ****P <0.0001.

To determine whether ACSL1 was regulated by STAT3 expression, we observed both mRNA and protein levels of ACSL1, together with other three STAT3-downstream genes (BCL2, c-MYC, CDKN1A) (35–37) in PRADX overexpressed, PRADX overexpressed plus WP1066(5µm) or DMSO-treated groups of N33 and N9 cells. The results exhibited that ACSL1, BCL2, c-MYC, and CDKN1A mRNA and protein expressions were increased in PRADX overexpressed tumor cells, in contrast to the PRADX overexpressed plus WP1066-treated group (**Figures 5A, B**), indicating PRADX-induced activation of STAT3 pathway and its downstream genes’ expression, including ACSL1. Then, we performed western blot and qRT-PCR analyses using N33 and N9 cells treated with different doses (1,2,5,10µM) of WP1066 (STAT3 inhibitor) or DMSO for 24h. The results showed that ACSL1 protein and mRNA levels were significantly reduced by WP1066 in a dose-dependent manner (**Figures 5C, D**), Furthermore, to determine whether ACSL1, BCL2, c-MYC, and CDKN1A expressions were regulated by PRADX through BLCAP suppression, we performed western blotting and qRT-PCR analyses Using N33 and N9 cells transfected with PRADX and BLCAP siRNA alone or in combination along with the negative control (NC) group. The results showed that ACSL1, BCL2, c-MYC, and CDKN1A expressions were reduced in the PRADX knockdown group. However, their expressions were increased in BLCAP with or without PRADX knockdown groups (**Figures 5** **E, F**). These data indicate PRADX overexpression can activate the STAT3 signaling pathway and its downstream genes *via* suppressing BLCAP expression.

### PRADX Overexpression Promotes Basal Respiration, Proton Leak, and ATP Production in GBM Cells *via* Increasing ACSL1

**Figure 6:**
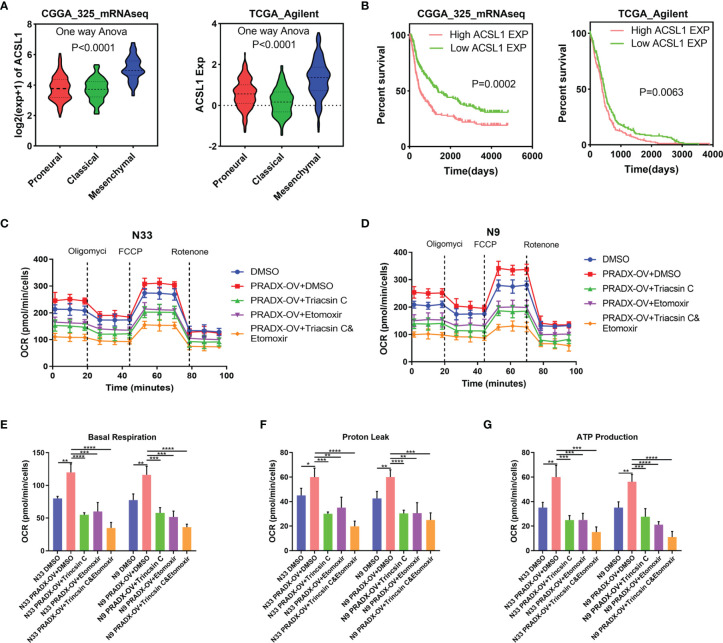
PRADX enhances energy metabolism *via* increasing ACSL1 in glioma. **(A)** Violin plots showing ACSL1 expression in different molecular subtypes of GBM in TCGA and CGGA databases. **(B)** Kaplan-Meier curve showing patients’ survival times between ACSL1 high expression and low expression groups. **(C, D)** The time series for the oxygen consumption rate (OCR) was measured by a seahorse analyzer in the indicated groups. **(E–G)** OCR measurement of basal respiration, proton leak, and ATP production for these five groups above. The values in **(C–G)** are represented as mean ± SD (n = 3). *P <0.05, **P < 0.01, ***P < 0.001, ****P <0.0001.

It has been reported that ACSL1 promotes tumorigenesis and development by regulating fatty acid metabolism (38). CPT1, located in the outer mitochondrial membrane, is the first rate-limiting factor in the mitochondrial fatty acid oxidation pathway. CPT1 plays a crucial role in transporting long-chain fatty acids and co-enzyme A into the mitochondrial matrix for β-oxidation (39). To examine whether the activation of ACSL1 by PRADX overexpression was correlated with mesenchymal GBM cell metabolism and ATP production, we explored the ACSL1 expression in TCGA and CGGA databases. One-way ANOVA analysis showed ACSL1 expression was significantly higher in the mesenchymal subtype than in proneural and classical subtypes of GBM (**Figure 6A**). Additionally, the Kaplan-Meier survival curve showed ACSL1 was associated with a shorter survival time in GBM patients (**Figure 6B**). Furthermore, we performed seahorse experiments using Triacsin C (ACSL1 inhibitor), and Etomoxir (CPT1 inhibitor) in PRADX overexpressed or scrambled N33 and N9 cells. The treated cells were divided into five groups, such as scramble plus DMSO, PRADX overexpressed, PRADX overexpressed plus Triacsin C (10µM), PRADX overexpressed plus Etomoxir (10µM), and PRADX overexpressed plus Triacsin C(10µM) plus Etomoxir(10µM). The oxygen consumption rate (OCR) represents real-time cellular respiration (**Figures 6C** **, D**). Basal respiration, proton leak, and ATP production were significantly increased in PRADX overexpressed groups. However, Triacsin C and Etomoxir reversed PRADX overexpression effects, as manifested by a reduction in basal respiration, proton leak, and ATP production levels in the two separate inhibitor groups. Additionally, these effects were even lower in the combination-treated groups (**Figures 6E** **–G**). These results demonstrated that PRADX overexpression increased cellular metabolism, and the combined application of Triacsin C and Etomoxir might be an efficient strategy to reverse PRADX-mediated metabolism acceleration.

### Combination of Triacsin C and Etomoxir Reverse PRADX-Mediated Tumor Cell Growth and Tumorigenesis

**Figure 7:**
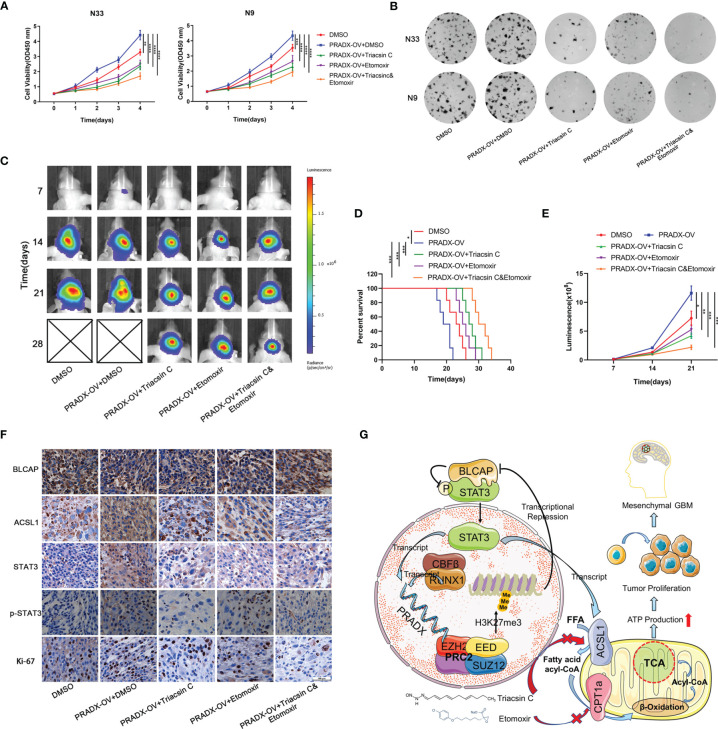
Triacsin C and Etomoxir reduce PRADX-mediated tumorigenesis *in vitro* and *in vivo*. **(A)** CCK8 assays show the effect of PRADX overexpression and the Triacsin C and Etomoxir on cell viability. **(B)** Colony formation assays show the effect of PRADX overexpression and the Triacsin C and Etomoxir on the tumor cell growth of the indicated five groups, representative images are shown. **(C)** Nude mice were orthotopically injected with PRADX overexpression or scramble TBD0220 cells. Representative luminescence images at 7,14,21 and 28d after injection are shown. **(D, E)** Kaplan-Meier survival curve and quantification of bioluminescence signal intensities at 7,14, and 21d post-injection are shown. **(F)** IHC of tumor tissues from nude mice xenograft models showing BLCAP, ACSL1, STAT3, p-STAT3, and Ki-67 expressions in the five groups. **(G)** Scheme showing PRADX-EZH2 interaction-mediated recruitment of H3K27me3 and regulation of STAT3 activity, promoting mesenchymal GBM tumorigenesis. The values in A are represented as mean ± SD (n = 3). *P < 0.05, **P < 0.01, ***P < 0.001, ****P <0.0001.

Cellular metabolism and ATP production are tightly associated with cell growth and tumorigenesis (40). CCK8 assay showed overexpressed PRADX could significantly promote the viability of N33 and N9 cells. However, treatments with Triacsin C (30µM) and Etomoxir (30µM) alone or in combination significantly inhibited cell viability in PRADX overexpressed tumor cells (**Figure 7A**). Furthermore, colony formation assays also demonstrated the inhibitory effects of Triacsin C (30uM), and Etomoxir (30uM) on tumor cell growth in PRADX overexpressed N33 and N9 cells (**Figure 7B**). To seek new treatment modalities for GBM patients with a high PRADX level *in vivo*, we constructed intracranial xenograft models with PRADX overexpressed TBD0220 cells and tested the efficiency of both the monotherapies and combination therapy with Triacsin C (30mg/kg/d) and Etomoxir (30mg/kg/d). Bioluminescence images captured at 7, 14, 21, and 28d post-implantation time-points showed significantly smaller tumors sizes in the combination therapy group (**Figure 7C**). Moreover, the Kaplan-Meier survival curve showed combination therapy group had the longest survival time among the treatment groups (**Figure 7D**). The quantification of luminescence further demonstrated that the combination therapy group had the smallest tumor volume (**Figure 7E**). Additionally, the IHC staining of the tumor sections showed the tumors from the combination therapy group had the lowest expression levels of ACSL1, STAT3, p-STAT3, and Ki-67. BLCAP expression in the combination group was the highest among the 5 groups (**Figure 7F**). The schematic diagram shows the PRADX-mediated recruitment of H3K27me3 and subsequent suppression of BLCAP expression, which in turn activates STAT3 and ACSL1 expressions, promoting mesenchymal GBM energy metabolism and tumorigenesis (**Figure 7G**).

## Discussion

Dysregulation of lncRNAs has consistently been discovered in a variety of cancers and multiple biological functions related to cancer progression (41). Studies have shown that lncRNA-mediated chromatin modification contributes to the malignant behavior of gliomas (12, 14, 42). Our previous studies have identified a novel lncRNA PRADX, which is overexpressed in GBM and COAD and correlates with the poor prognosis of GBM patients. PRADX promotes tumorigenesis *via* interacting with the PRC2 complex and recruiting H3K27me3 at the oncogene promoters, activating the NF-κB pathway (25). To further investigate the carcinogenic role of PRADX in GBM, we demonstrated PRADX transcription was modulated by the RUNX1-CBFβ complex. PRADX overexpression activated the STAT3 pathway, promoted cell metabolism, and ATP production *via* suppressing BLCAP expression. Our results suggested that the Combination treatment of ACSL1 and CPT1 inhibitors might serve as an effective treatment regimen for GBM.

Genome-wide mutation analysis revealed significant heterogeneity in GBM and led to three molecular subtypes (proneural, classical, and mesenchymal) to facilitate the exploration of GBM pathomechanism. Among the 3 types, mesenchymal GBM is the most malignant and usually presents with the worst prognosis (21, 27). Revealing the mechanism of mesenchymal GBM progression is essential for drug development and targeted therapy. In this study, we showed PRADX was highly expressed in mesenchymal GBM and transcriptionally regulated by the RUNX1-CBFβ complex. RUNX1 is a nuclear transcription factor, and CBFβ is necessary for RUNX1’s DNA binding affinity, but this mechanism has not been confirmed in solid tumors (28). Moreover, RUNX1 is a key mesenchymal GBM driver gene (23, 24). PARDX overexpression activates another two mesenchymal-specific genes like STAT3 and ACSL1. In addition, STAT3 also regulates PRADX mRNA expression (**Figure S2**). This might be an important regulatory feedback loop in the mesenchymal GBM development.

Several proteins function *via* interacting with other proteins. We performed co-IP and IF analyses and found out BLCAP non-functionally interacted with STAT3 and inhibited STAT3 phosphorylation. Overexpressed PRADX suppressed BLCAP expression *via* recruiting H3K27me3 at the BLCAP promoter, thus activating STAT3 phosphorylation. ACSL1 is mainly located in mitochondria, where it is involved in the biosynthesis of Acyl-COA that are channeled for subsequent β-Oxidation (43). Our results showed ACSL1 was reduced by WP1066 treatment in a dose-dependent manner, while PRADX overexpression accelerated cellular metabolism and ATP production *via* activating STAT3 phosphorylation and increasing ACSL1 expression. However, the mechanistic roles of ACSL1 in GBM energy metabolism and fatty acid production still need further exploration.

Acceleration of GBM cellular metabolic activity changes the phospholipid biosynthesis pathways, leading to energy metabolism reprogramming, which is an important feature of GBM (26, 44). Seeking clinical treatment strategies for GBM is the ultimate goal of our research. We showed that the combined use of Triacsin C and Etomoxir could reduce tumor cell metabolism and ATP production by seahorse experiment. Moreover, the combination of the ACSL1 and CPT1 inhibitors also reduced tumor growth both *in vivo* and *in vitro*. Our findings thus provide a potential therapeutic strategy for GBM treatment. However, the therapeutic efficiency of this treatment regimen in GBM patients needs further investigation. Additionally, investigating lncRNA-Protein interaction is also a potential therapeutic target for cancer therapy (25). Previous studies have focused on discovering EZH2 inhibitors for targeted therapy (17, 18), however, our study provided a novel therapeutic avenue for developing an effective inhibitor to disrupt PRADX-EZH2 interaction, thus increasing the expression of TSGs and finally inhibiting tumor growth.

In summary, our study revealed a novel lncRNA-protein interaction-mediated positive feedback loop which was important in mesenchymal GBM proliferation. PRADX/PRC2 complex recruited H3K27me3 at BLCAP promoter, suppressing BLCAP expression and activating BLCAP binding protein STAT3 phosphorylation, promoting ACSL1 expression and energy metabolism. Taken together, PRADX is a novel mesenchymal GBM biomarker, and RUNX1-CBFβ/PRADX/BLCAP/STAT3 axis is the key carcinogenic pathway in mesenchymal GBM, targeting ACSL1 and CPT1 to reduce cellular metabolism might be a potential strategy to treat GBM patients.

## Conclusion

We found the carcinogenic pathway of PRADX in mesenchymal GBM. PRADX was transcribed by the RUNX1-CBFβ complex. PRADX overexpression suppressed BLCAP expression *via* recruitment of H3K27me3, activating the phosphorylation of BLCAP interacting protein STAT3 and accelerating tumor metabolism and ATP production, resulting in tumor proliferation and poor survival outcomes.

## Data Availability Statement

The datasets presented in this study can be found in online repositories. The names of the repository/repositories and accession number(s) can be found in the article/**Supplementary Material**.

## Ethics Statement

The animal study was reviewed and approved by Institutional Committee on Animal Care of Hebei University (Approval No. IACUC-2021XG002).

## Author Contributions

Developed the study concept and designed the study: CK, CF, and YT. Conducted the experiments and acquired the data: CX, JZ, YT, JS, MX, XC, LX, JX, YZ, KY, BH, FT, and ST. Analyzed the data, generated the figures, and wrote the paper: CX and JZ. Funded the work and provided overall research supervision: CF and YT. Approved the final manuscript: All authors.

## Funding

This research was supported by grants from the National Natural Science Foundation of China (NSFC, No.82172660, No.82002657), Hebei Natural Science Foundation Precision Medicine Joint Project (No.H2020201206), Hebei provincial central leading local Science and Technology Development Fund Project (No.216Z7711G), Tianjin Key R&D Program of Tianjin Science and Technology Support Project (No.20YFZCSY00360), and the Science and Technology Project of Tianjin Municipal Health Commission (No.TJWJ2021QN003).

## Conflict of Interest

The authors declare that the research was conducted in the absence of any commercial or financial relationships that could be construed as a potential conflict of interest.

## Publisher’s Note

All claims expressed in this article are solely those of the authors and do not necessarily represent those of their affiliated organizations, or those of the publisher, the editors and the reviewers. Any product that may be evaluated in this article, or claim that may be made by its manufacturer, is not guaranteed or endorsed by the publisher.
