# Supplementary material for: lncRNA PRADX is a Mesenchymal Glioblastoma Biomarker for Cellular Metabolism Targeted Therapy
Source: Front Oncol. 2022 Apr 29;12:888922. doi: 10.3389/fonc.2022.888922 (PMC9106305; doi:10.3389/fonc.2022.888922)
Supplement: Supplementary file 1 [file DataSheet_1.docx]

**Supplementary materials**

**Supplementary Table 1**

**List of antibodies used for western blot, ChIP or co-IP**

| Antibody | Description |
| --- | --- |
| RUNX1 | Abcam (#ab272456) |
| CBFβ | Cell Signaling Technology (#62184) |
| BLCAP | Abcam (#ab129775) |
| STAT3 | Cell Signaling Technology (#12640) |
| p-STAT3 | Cell Signaling Technology (#9145) |
| BCL2 | Cell Signaling Technology (#4223) |
| c-MYC | Cell Signaling Technology (#18583) |
| CDKN1A | Cell Signaling Technology (#2947) |
| ACSL1 | Cell Signaling Technology (#9189) |

**Supplementary Table 2**

**List of Primers used for qRT-PCR**

|  | Forward Primer | Reverse Primer |
| --- | --- | --- |
| PRADX_1 | tcaaggggagaaggtaagcct | gaccccttgtacttgcctgat |
| PRADX_2 | ggtgtgaacccactccttgt | ggggcagggaagaggttttat |
| RUNX1 | ccacctaccacagagccatcaa | ttcactgagccgctcggaaaag |
| CBFβ | tgaagaggctcggagaaggaca | cgaagtttgaggtcatcaccacc |
| EZH2 | gacctctgtcttacttgtggagc | cgtcagatggtgtcacagcaatag |
| STAT3 | ctttgagaccgaggtgtatcacc | ggtcagcatgttgtaccacagg |
| ACSL1 | atcaggctgctcatggatgacc | agtccaagagccatcgcttcag |
| BCL2 | atcgccctgtggatgactgagt | gccaggagaaatcaaacagaggc |
| c-MYC | cctggtgctccatgaggagac | cagactctgaccttttgccagg |
| CDKN1A | aggtggacctggagactctcag | tcctcttggagaagatcagccg |
| BLCAP | ctgtggttcagccactccatgt | gaatcggagcagtggtacagga |
| GAPDH | ggtggtctcctctgacttcaaca | gttgctgtagccaaattcgttgt |

**Supplementary Table 3**

**List of primers used for ChIP**

|  | Forward primer | Reverse primer |
| --- | --- | --- |
| BLCAP#1 | aaggagtaggggcctaaggg | agagagagctggctttgcag |
| BLCAP#2 | tctgcaaagccagctctctc | ccaccatcctgctgcttaca |
| BLCAP#3 | tccaaacagctcgaaggagt | gccaccccagtatccaaca |
| BLCAP#4 | gccatcttcccaaggaggtt | cagtctaatggcgggtaggc |
| BLCAP#5 | cttctgtgctcacctcaccc | ggtggaacctccttgggaag |
| BLCAP#6 | acagacccaggttaccgact | tcaaatgcccaccttagggc |
| BLCAP#7 | tatgactgtctccccgccta | tactgtacccaagcgcagtg |
| BLCAP#8 | tttcagctcctgagccttgg | gttcatgttggtgaacccgc |
| BLCAP#9 | ttcgggcttgaagatctcgg | gcaaacgcctgatcacaagg |
| BLCAP#10 | gccgccatattgtcccttct | aatcacctgaagccggacag |
| PCDH10 | cccgtctacactgtgtccct | ggagtacacgacctcaccgt |
| PCDHB5 | aggtgtgtttgaccggagac | tccctatttcttcaccagcg |
| HOXD10 | ccgacaggcaggtcaagatt | tttccgctttcccagtcctc |
| RUNX1#1 | cgtaggtgctggacttgtcg | gcgggctccatggtagattg |
| RUNX1#2 | aggtgctggacttgtcgc | cgggctccatggtagattgta |
| RUNX1#3 | agccatagtggatgtcaaacga | gtgcgggctccatggtagat |

**Supplementary Table 4**

**List of siRNAs used for cell transfection**

|  | sense | antisense |
| --- | --- | --- |
| siRUNX1#1 | ucacugugauggcuggcaatt | uugccagccaucacagugatt |
| siRUNX1#2 | ccagguugcaagauuuaautt | auuaaaucuugcaaccuggtt |
| siRUNX1#3 | gcuucacucugaccaucactt | gugauggucagagugaagctt |
| siCBFβ#1 | gaagcaaguucgagaacgatt | ucguucucgaacuugcuuctt |
| siCBFβ#2 | caggaaccaaucugucucutt | agagacagauugguuccugtt |
| siCBFβ#3 | caggcaagguauauuugaatt | uucaaauauaccuugccugtt |
| siEZH2#1 | ggaucaccgagaugauaaatt | uuuaucaucucggugaucctt |
| siEZH2#2 | gcuccucuaaccauguuuatt | uaaacaugguuagaggagctt |
| siEZH2#3 | gagggaaaguguaugauaatt | uuaucauacacuuucccuctt |
| siBLCAP#1 | gguucagccacuccauguutt | aacauggaguggcugaacctt |
| siBLCAP#2 | gcagcccuguuccuuaucutt | agauaaggaacagggcugctt |
| siBLCAP#3 | gcuuccagaaucggcgcautt | augcgccgauucuggaagctt |
| siPRADX#1 | gcacaggccacaguccuaatt | uuaggacuguggccugugctt |
| siPRADX#2 | gcaccugcgcuggucucuutt | aagagaccagcgcaggugctt |
| siPRADX#3 | ccugucacuccugcccaaatt | uuugggcaggagugacaggtt |
| NC | uucuccgaacgugucacgutt | acgugacacguucggagaatt |

**Supplementary Figures**

**
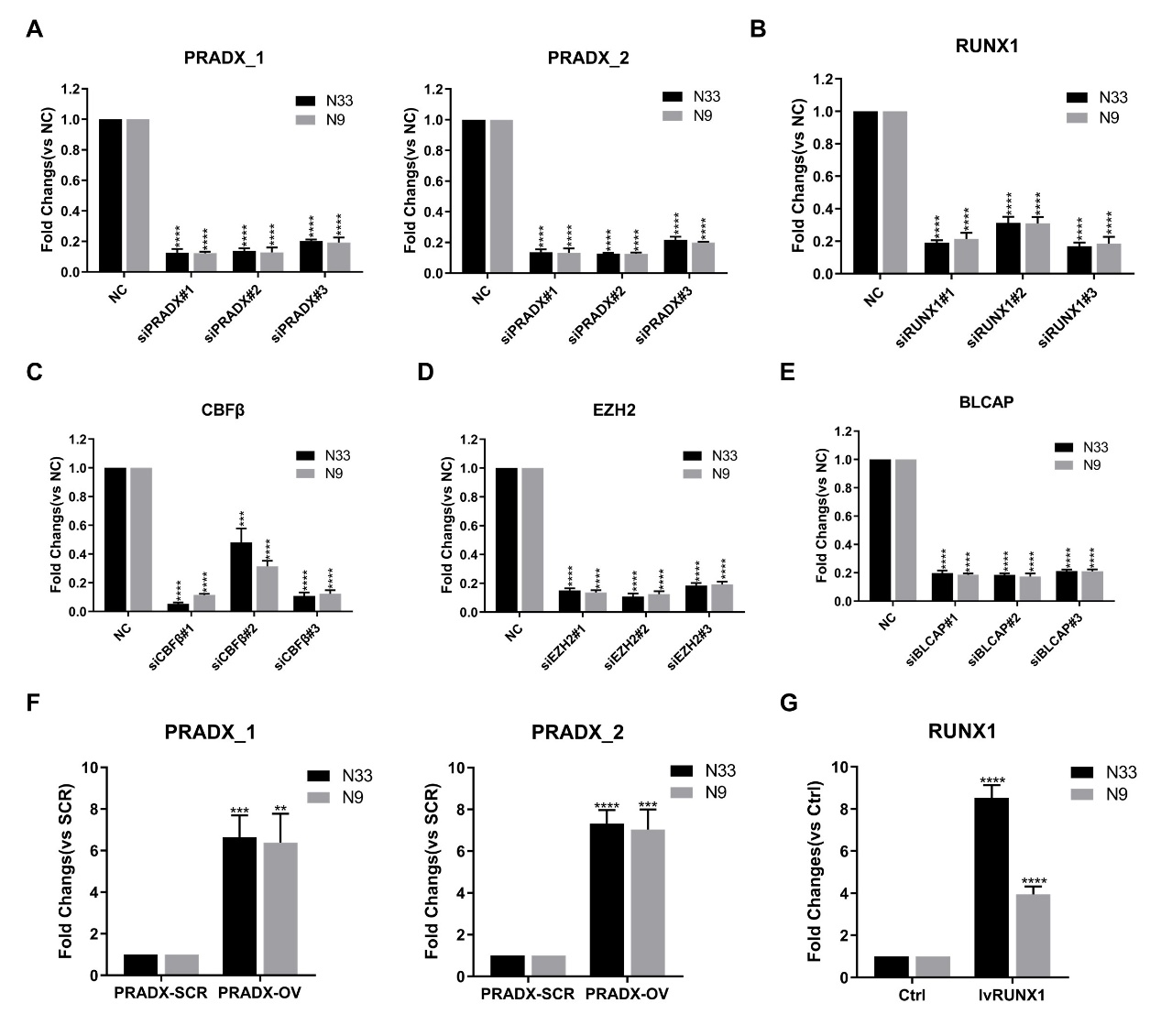
**

**Supplementary Figure 1.** A-E. Transfection efficiency of siRNA targeting PRADX, RUNX1, CBFβ, EZH2, or BLCAP in N33 and N9 cells. F. Transfection efficiency of PRADX lentivirus in N33 and N9 cells. G. Transfection efficiency of RUNX1 expression plasmid in N33 and N9 cells.

**
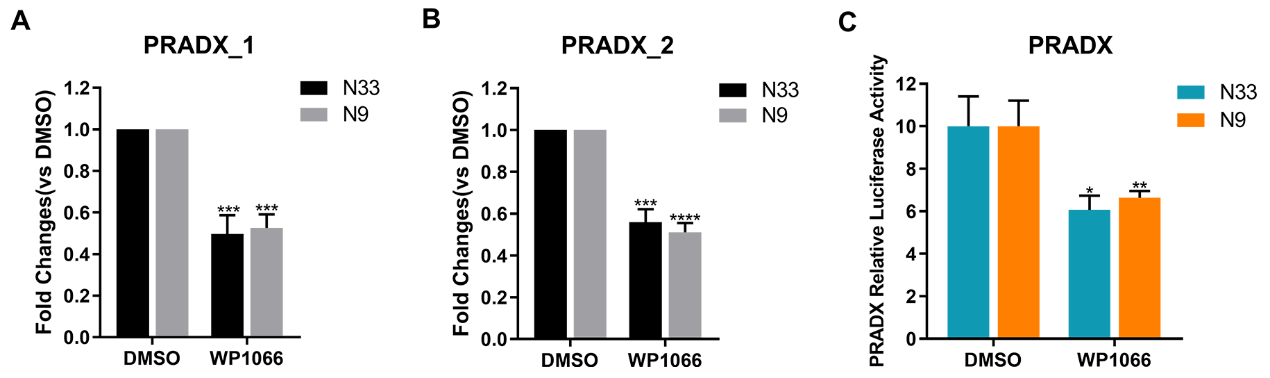
**

**Supplementary Figure 2.** A-B. qRT-PCR analysis showing Relative PRADX expression after treated with WP1066 (5µm). C. Dual-luciferase reporter assay exhibiting PRADX relative luciferase activity after treated with WP1066 (5µm).
